# Supplementary material for: Dynamics of microcompartment formation at the mitosis-to-G1 transition
Source: Nat Struct Mol Biol. 2025 Oct 17;32(12):2614–27. doi: 10.1038/s41594-025-01687-2 (PMC12700819; doi:10.1038/s41594-025-01687-2)
Supplement: Supplementary file 1 — Supplementary Methods, Figs. 1–15, Tables 1–3 and References. [file 41594_2025_1687_MOESM1_ESM.pdf]

nature structural & molecular biology

Supplementary information https://doi.org/10.1038/s41594-025-01687-2

Dynamics of microcompartment formation at the mitosis-to-G1 transition

In the format provided by the authors and unedited

Supplementary Information for

Dynamics of microcompartment formation at the mitosis-to-G1 transition

Viraat Y. Goel[1-4], Nicholas G. Aboreden[5,6], James M. Jusuf[1-4], Haoyue Zhang[7], Luisa P. Mori[6], Leonid A. Mirny[8], Gerd A. Blobel[5,6], Edward J. Banigan[8,*], Anders S. Hansen[1-4,*]

[1] Department of Biological Engineering, Massachusetts Institute of Technology; Cambridge, MA 02139, USA
[2] Gene Regulation Observatory, Broad Institute of MIT and Harvard; Cambridge, MA 02142, USA
[3]The Novo Nordisk Foundation Center for Genomic Mechanisms of Disease, Broad Institute of MIT and Harvard, Cambridge, MA 02142, USA
[4] Koch Institute for Integrative Cancer Research; Cambridge, MA, 02139, USA
[5] Perelman School of Medicine, University of Pennsylvania, Philadelphia, PA, USA
[6] Division of Hematology, The Children's Hospital of Philadelphia, Philadelphia, PA, USA
[7] Institute of Molecular Physiology, Shenzhen Bay Laboratory, Shenzhen, Guangdong, China
[8] Institute for Medical Engineering and Science and Department of Physics, Massachusetts Institute of Technology, Cambridge, 02139 MA, USA
* Co-corresponding authors: EJB ebanigan@mit.edu and ASH ashansen@mit.edu

Table of Contents

SUPPLEMENTARY METHODS 2
SUPPLEMENTARY TABLES 5
SUPPLEMENTARY FIGURES 7
SUPPLEMENTARY INFORMATION REFERENCES 22

Supplementary Methods

Summary of key steps in the Region Capture Micro-C Protocol

Crosslinking

Crosslinking was performed precisely as in Goel *et al.* 2023[1]. Briefly, cells were doubly crosslinked using 3 mM DSG (ThermoFisher #20593) and 1% formaldehyde (ThermoFisher #28906). Single cells were counted, washed in PBS, and then resuspended in 3mM DSG in PBS at a concentration of 1M cells per mL, mixed gently for 35 min at room temperature after which formaldehyde was added to a final concentration of 1% and gently mixed at room temperature for an additional 10 minutes and finally quenched with Tris buffer pH = 7.5 (K-D Medical #RGE-3370) at a final concentration of 0.375 M. Crosslinked cells were washed twice with 1X PBS, re-counted, and then partitioned into 1-5M cell aliquots that were pelleted and snap-frozen in liquid nitrogen for storage at –80°C.

Micrococcal nuclease (MNase) titration

MNase titration was performed as described in Goel *et al.* 2023. Ideal digestion conditions were determined for each batch of crosslinked cells by treating 1M cell samples with varying amounts of MNase and digesting at 37°C for 20 min on a thermomixer. Digested chromatin underwent crosslink reversal, DNA purification, and gel-based separation to visualize the fragment size distribution. Ideal digestion concentrations were identified by samples digested to primarily (~80%) monomeric fragments (150-200 bp), few (~15-20%) dimeric fragments (250-350 bp), and a faint but visible band (<5%) of trimeric fragments (400-500 bp).

Sample inputs

RCMC was performed on cellular inputs ranging from 1-25M of total post-crosslinking sample for each sample condition and replicate. For samples with ample crosslinked material, multiple 5M cell samples were carried in parallel through the protocol to maximize library complexity and combined into a single tube during library preparation. For scarcer samples (e.g., 5M cells or less), RCMC was performed on all available material in a single tube. All M-to-G1 RCMC datasets in this manuscript were generated from two biological replicates of 15-25M input cells each. The condensin degron RCMC datasets in this manuscript were generated from 2-4 biological replicates of 0.2-5M input cells each, with significantly less crosslinked material available for the 0.5h and 8h auxin treatment conditions than for the 0h, 1h, and 4h conditions.

Micrococcal nuclease digestion

As described in Goel *et al.* 2023, cell membranes were solubilized to extract intact nuclei by resuspending crosslinked 5M cell pellets in Micro-C Buffer #1 (MB#1; 50 mM NaCl, 10 mM Tris-HCl pH = 7.5, 5 mM MgCl2, 1M CaCl2, 0.2% NP-40 Alternative (Millipore Sigma #492018), 1x Protease Inhibitor Cocktail (Sigma-Aldrich #5056489001)) at 1M cells per 100 µL for 20 min on ice. Following an MB#1 wash, samples were resuspended in 100 µL MB#1 and the ideal amount of 20 U/µL MNase (Worthington Biochem #LS004798) determined by the MNase titration was added. This digestion reaction was mixed at 37°C for 20 min on a thermomixer before being quenched with 4 mM EGTA (bioWORLD #40520008) and heat inactivated at 65°C for 10 min. Digested nuclei were washed twice with ice-cold Micro-C Buffer #2 (50 mM NaCl, 10 mM Tris-HCl pH = 7.5, 10 mM MgCl2, 100 µg/mL BSA (Sigma-Aldrich #B8667)).

End repair and labeling

As described in Goel *et al.* 2023, digested fragments were enzymatically blunted and biotinylated. First, digested chromatin was 5' phosphorylated in end-repair reactions (50 U T4 Polynucleotide Kinase (New England BioLabs #M0201), 50 mM NaCl, 10 mM Tris-HCl pH = 7.5, 10 mM MgCl2, 100 ug/mL BSA, 2 mM ATP (ThermoFisher #R1441), 5 mM DTT (Sigma-Aldrich #10197777001), in water) at 37°C for 15 min while mixing. Next, 50 U of DNA Polymerase I Klenow Fragment (New England BioLabs #M0210) was added to the reaction and incubated at 37°C for 15 min while mixing to create 5' fragment overhangs, and these overhangs were filled in by adding a mixture of dNTPs in end-labelling buffer (66 µM each of dTTP (Jena Bioscience #NU-1004), dGTP (Jena Bioscience #NU-1003), biotin-dATP (Jena Bioscience #NU-835-BIO14), and biotin-CTP (Jena Bioscience #NU-809-BIOX), 1X T4 DNA Ligase Buffer, 100 µg/mL BSA, in water) and incubating at room temperature for 45 min with interval mixing. This end-blunting reaction was quenched by 30 mM EDTA (Invitrogen #15575020) and heat inactivated at 65°C for 20 min. Finally, end-blunted and biotin-labeled nuclei were washed once with Micro-C Buffer #3 (50 mM Tris-HCl pH = 7.5, 10 mM MgCl2, 100 µg/mL BSA).

Proximity ligation and removal of unligated biotin

As described in Goel *et al.* 2023, proximity ligation was performed by incubating labeled chromatin in a ligation reaction (10,000 U T4 DNA Ligase (New England BioLabs #M0202), 1X T4 DNA Ligase Buffer, 100 µg/mL BSA, in 500 µL water) at room temperature for at least 2.5 hours or overnight with gentle mixing. To remove biotinylated dNTPs from all unligated fragment ends, samples were digested by 1,000 U of Exonuclease III (New England BioLabs #M0206) in reaction buffer (1X NEBuffer #1 in water) at 37°C for 15 min with interval mixing.

DNA purification and size-selection

As described in Goel *et al.* 2023, ligated DNA fragments were purified over a series of steps. DNA was first reverse crosslinked to remove proteins and RNA by adding 1% SDS (Sigma-Aldrich #L3771), 2 mg/mL Proteinase K (Viagen Biotech #501-PK), 250 mM NaCl, and 100 µg/mL RNaseA (ThermoFisher #EN0531) to the samples and incubating at 65°C overnight. Following crosslink reversal, the DNA solution was purified using the Zymo DNA Clean & Concentrator kit (Zymo Research #D4034) according to the kit manual.

Ligated DNA fragments were subsequently size-selected (~200-400 bp) by extraction from a 1% agarose gel (VWR #97062). Gel extracts were purified using the Zymo Gel Purification kit (Zymo Research #D4008), and samples were quantified by Qubit 1X dsDNA High Sensitivity Assay (Invitrogen #Q33231).

Ligated fragments were further purified by using Dynabeads MyOne Streptavidin T1 (Invitrogen #65601) to enrich for biotinylated fragments. DNA samples were bound to beads in a Binding and Wash Buffer (1 M NaCl, 5 mM Tris-HCl pH = 7.5, 500 µM EDTA, 0.1% Tween-20 (Sigma-Aldrich #P8074)) at room temperature for at least 30 minutes with mixing. After two washes with the Binding and Wash Buffer, the bead-bound samples were washed once with 10 mM Tris-HCl pH = 7.5 prior to library prep.

Library preparation

As described in Goel *et al.* 2023, Illumina library preparation was performed using the NEBNext Ultra II kit (New England BioLabs #E7645), with the addition of interval shaking (1 minute on, 3 minutes off) at 1000 rpm during incubations to mix the bead-bound samples. Sample washes were performed using Binding and Wash Buffer and 10 mM Tris-HCl pH = 7.5. A test library amplification determined the number of PCR cycles necessary to meet Capture input requirements (200-500 ng per sample) using 5% or less of the prepped library, with test PCR reactions run on an agarose gel and yields quantified using image quantification software Image Studio Lite (LI-COR Biosciences). The M-to-G1 RCMC replicates in this manuscript used 6-9 PCR cycles for final library amplification while the condensin degron RCMC replicates ranged from 7-17 cycles, with samples having RCMC inputs below 5M cells (e.g., all 0.5h and 8h replicates) requiring more cycles. Libraries were separately indexed by sample and replicate using sequencing indices from the NEB Multiplex Oligos for Illumina Primer Sets 1 and 2 (New England BioLabs #E7335 and #E7500), and amplification was done using the KAPA HiFi HotStart ReadyMix (Roche #07958927001). Following library amplification, amplified libraries were purified to remove adaptor dimers, primers, and contaminants using AmPure XP beads (Beckman Coulter #A63880). Purified libraries were quantified via Fragment Analyzer and qPCR at the MIT BioMicro Center to determine library concentrations for pooling prior to Capture.

Capture probe design

As described in Goel *et al.* 2023, target loci of interest were identified based on genomic features or enhancer-promoter relationships of interest (**Extended Data Fig. 1b**). The *Klf1* locus, which we previously reported[1], was selected for its dense microcompartments. The *Dag1*, *Id1*, and *Cdt1* loci were selected as similarly gene-rich loci likely to exhibit a mixture of microcompartment, A/B compartment, and CTCF loop features while also containing genes relevant to cell cycle control. The *Myc* locus, heavily studied for MYC's role as a key transcription factor associated with disease and cell cycle control, was selected as a relatively gene-poor control with a well-characterized function and regulatory relationships. Using the UCSC Genome Browser and HiGlass visualization of existing G1E-ER4 Hi-C datasets, locus bounds were selected to include visible local structures and genomic features in roughly 1-2 Mb-sized regions. Once loci had been selected, 80-mer probes were designed to tile end-to-end without overlap across the Capture loci through Twist Bioscience. Probes with high predicted likelihoods of off-target pulldown (e.g., such as those in high-repeat regions) were masked and removed from the probe tiling, and probe coverage was double-checked to ensure the inclusion of key genomic features (e.g., all promoters and CTCF sites in the locus) before finalization. Probe panels were synthesized and purchased as Custom Target Enrichment Panels from Twist Bioscience.

Capture of target loci

As described in Goel *et al.* 2023, Capture was performed in accordance with Twist Bioscience's Standard Hybridization Target Enrichment Protocol. Sample libraries were pooled in a 1:1 molar ratio across conditions, after which they were dried and mixed with Hybridization Mix (Twist Bioscience #104178), Custom Panels (Twist Bioscience #101001), Universal Blockers (Twist Bioscience #100578), and Mouse Cot-1 DNA (Invitrogen #18440016). The library pool was hybridized to the biotinylated probe panel overnight, after which streptavidin beads (Twist Bioscience #100983) were used to pull down probes with hybridized ligated fragments and then washed (Twist Bioscience #104178) to remove unbound fragments. Another round of PCR amplified the target-enriched library using the Equinox Library Amplification Mix (Twist Bioscience #104178), with a test PCR included (as described above) to identify the number of amplification cycles necessary to meet sequencing requirements. With 2-4 µg of pooled input library for Capture, the RCMC samples generated in this manuscript needed 4-5 cycles of post-Capture PCR amplification. Following PCR amplification, the Captured library was purified (Twist Bioscience #100983) and then quantified via both Fragment Analyzer and qPCR at the MIT BioMicro Center in preparation for sequencing submission.

Sequencing

Following qPCR quantification, post-Capture libraries across replicates were pooled in a 1:1 molar ratio. Pooled libraries were paired-end sequenced using 2x50 cycle sequencing kits with Illumina NovaSeq S1 flow cells on a NovaSeq 6000 system (only Biological Replicate 1 of the M-to-G1 samples) or using 2x150 cycle sequencing kits on a NovaSeq X system (all biological replicates across all conditions) by the Broad Institute of MIT and Harvard's Walk-Up Sequencing services. Basecalls for NovaSeq output were performed using bcl2fastq v2.20.0.422.

Supplementary Tables

Supplementary Table 1. List of published datasets used in this paper.

This table contains a list of public datasets used in this paper for the cell line G1E-ER4.

	Condition	GEO / ENCODE#	Reference
CTCF ChIP-Seq	prometaphase	GSE129997	Zhang, H., Emerson, D.J., Gilgenast, T.G. et al. Chromatin structure dynamics during the mitosis-to-G1 phase transition. Nature 576, 158–162 (2019). https://doi.org/10.1038/s41586-019-1778-y
CTCF ChIP-Seq	ana/telophase	GSE129997	Zhang, H., Emerson, D.J., Gilgenast, T.G. et al. Chromatin structure dynamics during the mitosis-to-G1 phase transition. Nature 576, 158–162 (2019). https://doi.org/10.1038/s41586-019-1778-y
CTCF ChIP-Seq	early G1	GSE129997	Zhang, H., Emerson, D.J., Gilgenast, T.G. et al. Chromatin structure dynamics during the mitosis-to-G1 phase transition. Nature 576, 158–162 (2019). https://doi.org/10.1038/s41586-019-1778-y
CTCF ChIP-Seq	mid G1	GSE129997	Zhang, H., Emerson, D.J., Gilgenast, T.G. et al. Chromatin structure dynamics during the mitosis-to-G1 phase transition. Nature 576, 158–162 (2019). https://doi.org/10.1038/s41586-019-1778-y
CTCF ChIP-Seq	late G1	GSE129997	Zhang, H., Emerson, D.J., Gilgenast, T.G. et al. Chromatin structure dynamics during the mitosis-to-G1 phase transition. Nature 576, 158–162 (2019). https://doi.org/10.1038/s41586-019-1778-y
CTCF ChIP-Seq	asynchronous	GSE129997	Zhang, H., Emerson, D.J., Gilgenast, T.G. et al. Chromatin structure dynamics during the mitosis-to-G1 phase transition. Nature 576, 158–162 (2019). https://doi.org/10.1038/s41586-019-1778-y
RAD21 (cohesin) ChIP-Seq	prometaphase	GSE129997	Zhang, H., Emerson, D.J., Gilgenast, T.G. et al. Chromatin structure dynamics during the mitosis-to-G1 phase transition. Nature 576, 158–162 (2019). https://doi.org/10.1038/s41586-019-1778-y
RAD21 (cohesin) ChIP-Seq	ana/telophase	GSE129997	Zhang, H., Emerson, D.J., Gilgenast, T.G. et al. Chromatin structure dynamics during the mitosis-to-G1 phase transition. Nature 576, 158–162 (2019). https://doi.org/10.1038/s41586-019-1778-y
RAD21 (cohesin) ChIP-Seq	early G1	GSE129997	Zhang, H., Emerson, D.J., Gilgenast, T.G. et al. Chromatin structure dynamics during the mitosis-to-G1 phase transition. Nature 576, 158–162 (2019). https://doi.org/10.1038/s41586-019-1778-y
RAD21 (cohesin) ChIP-Seq	mid G1	GSE129997	Zhang, H., Emerson, D.J., Gilgenast, T.G. et al. Chromatin structure dynamics during the mitosis-to-G1 phase transition. Nature 576, 158–162 (2019). https://doi.org/10.1038/s41586-019-1778-y
RAD21 (cohesin) ChIP-Seq	late G1	GSE129997	Zhang, H., Emerson, D.J., Gilgenast, T.G. et al. Chromatin structure dynamics during the mitosis-to-G1 phase transition. Nature 576, 158–162 (2019). https://doi.org/10.1038/s41586-019-1778-y
RAD21 (cohesin) ChIP-Seq	asynchronous	GSE129997	Zhang, H., Emerson, D.J., Gilgenast, T.G. et al. Chromatin structure dynamics during the mitosis-to-G1 phase transition. Nature 576, 158–162 (2019). https://doi.org/10.1038/s41586-019-1778-y
RNA Pol II ChIP-Seq	prometaphase	GSE129997	Zhang, H., Emerson, D.J., Gilgenast, T.G. et al. Chromatin structure dynamics during the mitosis-to-G1 phase transition. Nature 576, 158–162 (2019). https://doi.org/10.1038/s41586-019-1778-y
RNA Pol II ChIP-Seq	ana/telophase	GSE129997	Zhang, H., Emerson, D.J., Gilgenast, T.G. et al. Chromatin structure dynamics during the mitosis-to-G1 phase transition. Nature 576, 158–162 (2019). https://doi.org/10.1038/s41586-019-1778-y
RNA Pol II ChIP-Seq	early G1	GSE129997	Zhang, H., Emerson, D.J., Gilgenast, T.G. et al. Chromatin structure dynamics during the mitosis-to-G1 phase transition. Nature 576, 158–162 (2019). https://doi.org/10.1038/s41586-019-1778-y
RNA Pol II ChIP-Seq	mid G1	GSE129997	Zhang, H., Emerson, D.J., Gilgenast, T.G. et al. Chromatin structure dynamics during the mitosis-to-G1 phase transition. Nature 576, 158–162 (2019). https://doi.org/10.1038/s41586-019-1778-y
RNA Pol II ChIP-Seq	late G1	GSE129997	Zhang, H., Emerson, D.J., Gilgenast, T.G. et al. Chromatin structure dynamics during the mitosis-to-G1 phase transition. Nature 576, 158–162 (2019). https://doi.org/10.1038/s41586-019-1778-y
H3K4me1 ChIP-Seq	asynchronous	GSM946535	Mouse ENCODE Consortium., Stamatoyannopoulos, J.A., Snyder, M. et al. An encyclopedia of mouse DNA elements (Mouse ENCODE). Genome Biol 13, 418 (2012). https://doi.org/10.1186/gb-2012-13-8-418
H3K4me3 ChIP-Seq	asynchronous	ENCFF098DTA	Mouse ENCODE Consortium., Stamatoyannopoulos, J.A., Snyder, M. et al. An encyclopedia of mouse DNA elements (Mouse ENCODE). Genome Biol 13, 418 (2012). https://doi.org/10.1186/gb-2012-13-8-418
H3K27ac ChIP-Seq	asynchronous	GSE61349	Dogan N, Wu W, Morrissey CS, Chen KB et al. Occupancy by key transcription factors is a more accurate predictor of enhancer activity than histone modifications or chromatin accessibility. Epigenetics Chromatin 2015;8:16. PMID: 25984238
H3K9me3 ChIP-Seq	asynchronous	ENCFF480HQQ	Mouse ENCODE Consortium., Stamatoyannopoulos, J.A., Snyder, M. et al. An encyclopedia of mouse DNA elements (Mouse ENCODE). Genome Biol 13, 418 (2012). https://doi.org/10.1186/gb-2012-13-8-418
H3K27me3 ChIP-Seq	asynchronous	ENCFF978WJA	Mouse ENCODE Consortium., Stamatoyannopoulos, J.A., Snyder, M. et al. An encyclopedia of mouse DNA elements (Mouse ENCODE). Genome Biol 13, 418 (2012). https://doi.org/10.1186/gb-2012-13-8-418
H3K36me3	asynchronous	ENCFF452ACX	Mouse ENCODE Consortium., Stamatoyannopoulos, J.A., Snyder, M. et al. An encyclopedia of mouse DNA elements (Mouse ENCODE). Genome Biol 13, 418 (2012). https://doi.org/10.1186/gb-2012-13-8-418

Supplementary Table 2. Default parameters in parameter sweeps.

This table contains the default parameters used in the sweeps for the polymer simulations.

Parameter	Value	Notes & References
Loop extruder residence time, τ_{res}	500 s	Within observed range for condensin and cohesin[2–10].
Loop extruder linear density, $1/d$	1 / 100 kb	Within observed ranges[2,11–13].
Loop extruder speed, v	1 kb/s	Based on *in vitro* studies[14–18].
Probability of cohesin stopping at CTCF, q	0	CTCF ignored for parameter sweeps.
Monomer/lattice site genomic size	0.5 kb	Selected for resolution of microcompartments.

Monomer physical size	25 nm	Calculated as described in Methods and Gabriele *et al.* 2022[2].
Chromosome length	30.3 Mb	Practical decision.
Rouse scaling prefactor for 515 kb segment, Γ	0.0076 $\mu m^2/s^{1/2}$	Gabriele *et al.* 2022[2].
A-type compartment affinity	0 k_BT	Conventional selection (e.g. see Ref [19,20])
B-type compartment affinity	0.05 k_BT	Selected by parameter sweep
C-type (micro)compartment affinity	0.9 k_BT	Selected by parameter sweep
Chromatin volume fraction, ρ_{chr}	0.25	Ou *et al.* 2017[21].

Supplementary Table 3. Default parameters in mitosis-to-G1 transition simulations.
This table contains the default parameters for the polymer simulations that run from prometaphase to late G1 phase.

Parameter	Value	Notes & References
Condensin I residence time, τ_{res}^{CI}	3 min	Based on FRAP[4,5].
Condensin II residence time, τ_{res}^{CII}	1 h	Models stably bound condensin II observed in experiments[4,5] and similar to previous simulations[22].
Cohesin residence time, $\tau_{res}^{cohesin}$	10 min	Based on FRAP studies[3,7–10].
Condensin I linear density in prometaphase, $1/d^{CI}$	1 / 35 kb	Within observed ranges[5,23–26].
Peak condensin I linear density prior to ana/telophase, $1/d^{CI,*}$	1 / 27 kb	Estimated from Ref [4,5].
Condensin II linear density, d^{CII}	1 / 140 kb	Within observed ranges[5,23–26].
Cohesin linear density, $d^{cohesin}$	1 / 100 kb	Within observed ranges[2,11–13].
Loop extruder speed, v	1 kb/s	Based on *in vitro* studies[14–18].
Probability of cohesin stopping at CTCF, q	0.5	Selected by parameter sweep
Monomer/lattice site genomic size	0.5 kb	Selected for resolution of microcompartments
Monomer physical size	25 nm	Calculated as described in Methods and Gabriele *et al.* 2022[2].
Chromosome length	30.3 Mb	Practical decision
Rouse scaling prefactor for 515 kb segment, Γ	0.0076 $\mu m^2/s^{1/2}$	Gabriele *et al.* 2022[2].
A-type compartment affinity	0 k_BT	Conventional selection (e.g. see Ref [19,20])
B-type compartment affinity	0.05 k_BT	Selected by parameter sweep
C-type (micro)compartment affinity	0.9 k_BT	Selected by parameter sweep
Interphase chromatin density, ρ_{chr}	0.25	Ou *et al.* 2017[21].
Mitotic chromatin density, ρ_{chr}	0.65	Between previous experimental[21,27,28] and simulated values[29].
Prometaphase cylindrical confinement aspect ratio	4	Consistent with observations across literature

Supplementary Figures

a Contact maps reveal non-DNase microcompartment anchors and DNase peaks that do not form microcompartments

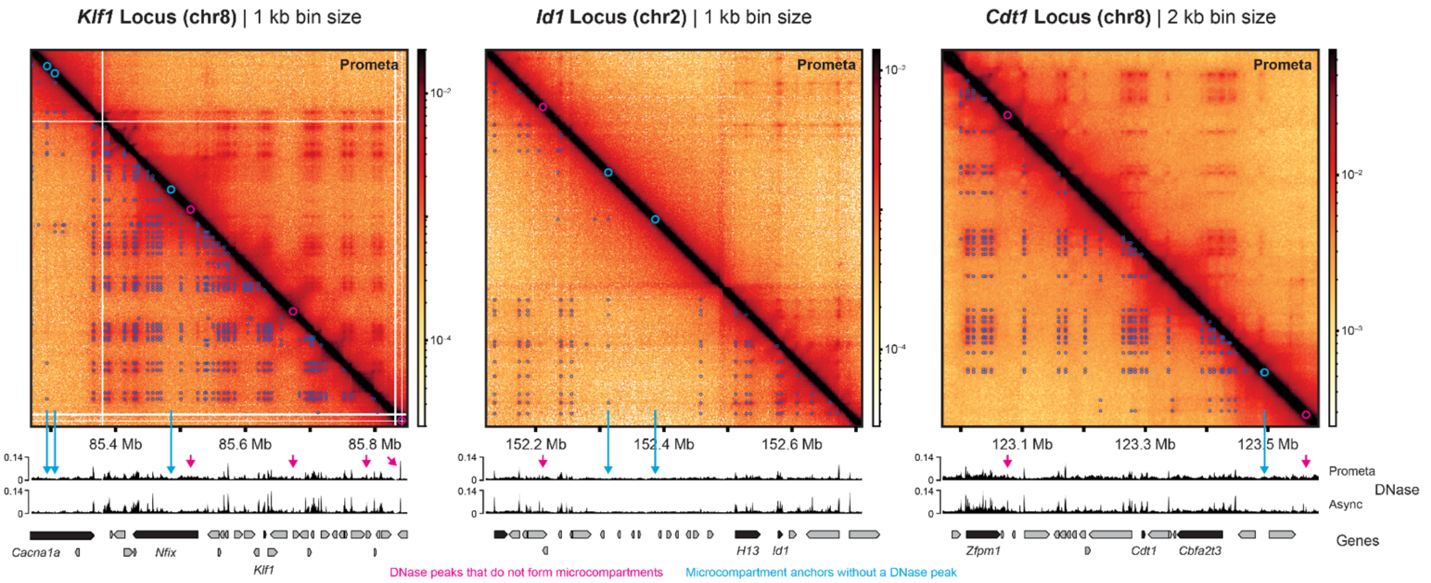

b Venn diagram relationship between DNase peaks & microcompartment anchors

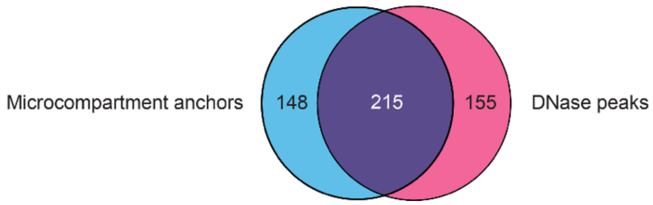

Supplementary Figure 1. Microcompartments are not artifacts of chromatin accessibility. (**a**) RCMC contact maps at the *Klf1* (left, 1 kb resolution), *Id1* (middle, 1 kb resolution), and *Cdt1* (right, 1 kb resolution) loci indicating DNase-Seq peaks that do not form microcompartments (magenta) and microcompartment anchors that do not coincide with a DNase peak (cyan). (**b**) Venn diagram breakdown of the overlap between all annotated microcompartment anchors and all DNase peaks across captured loci. Of 363 annotated microcompartment anchors, 148 do not coincide with DNase peaks (cyan) while 215 do (purple). Of 370 called DNase peaks, 155 do not form microcompartment anchors (magenta) while 215 do (purple).

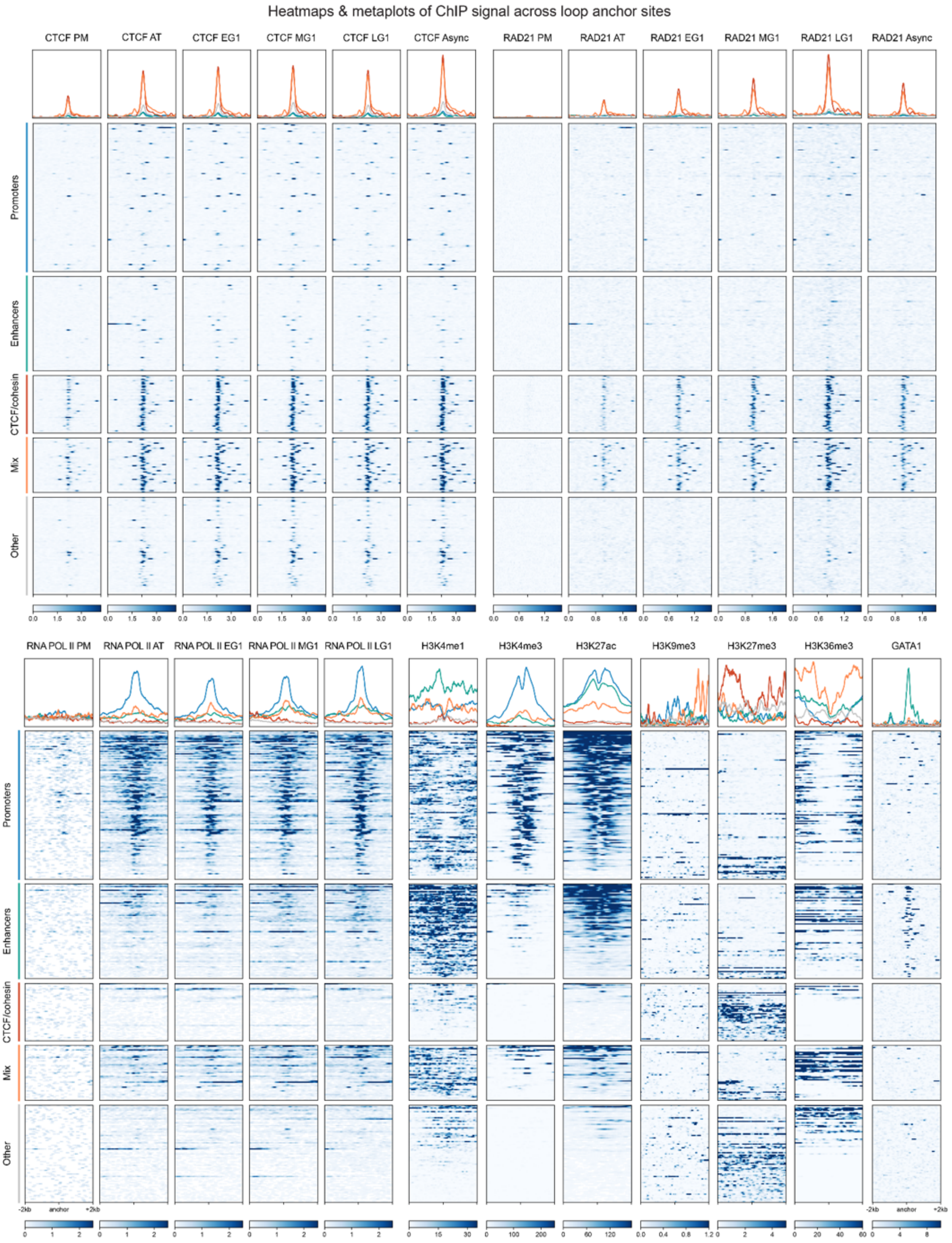

Supplementary Figure 2. Categories of microcompartment anchors can be defined by their chromatin features. Metaplots (above) and heatmaps (below) depicting ChIP-seq (Supplementary Table 1) signal at annotated loop anchors as defined in Fig. 2d, including promoters (blue), enhancers (green), CTCF and RAD21-bound (red), a mix of both a cis-regulatory element and CTCF/cohesin (orange), and other (gray). Features are plotted in a 2 kb window centered on the anchor.

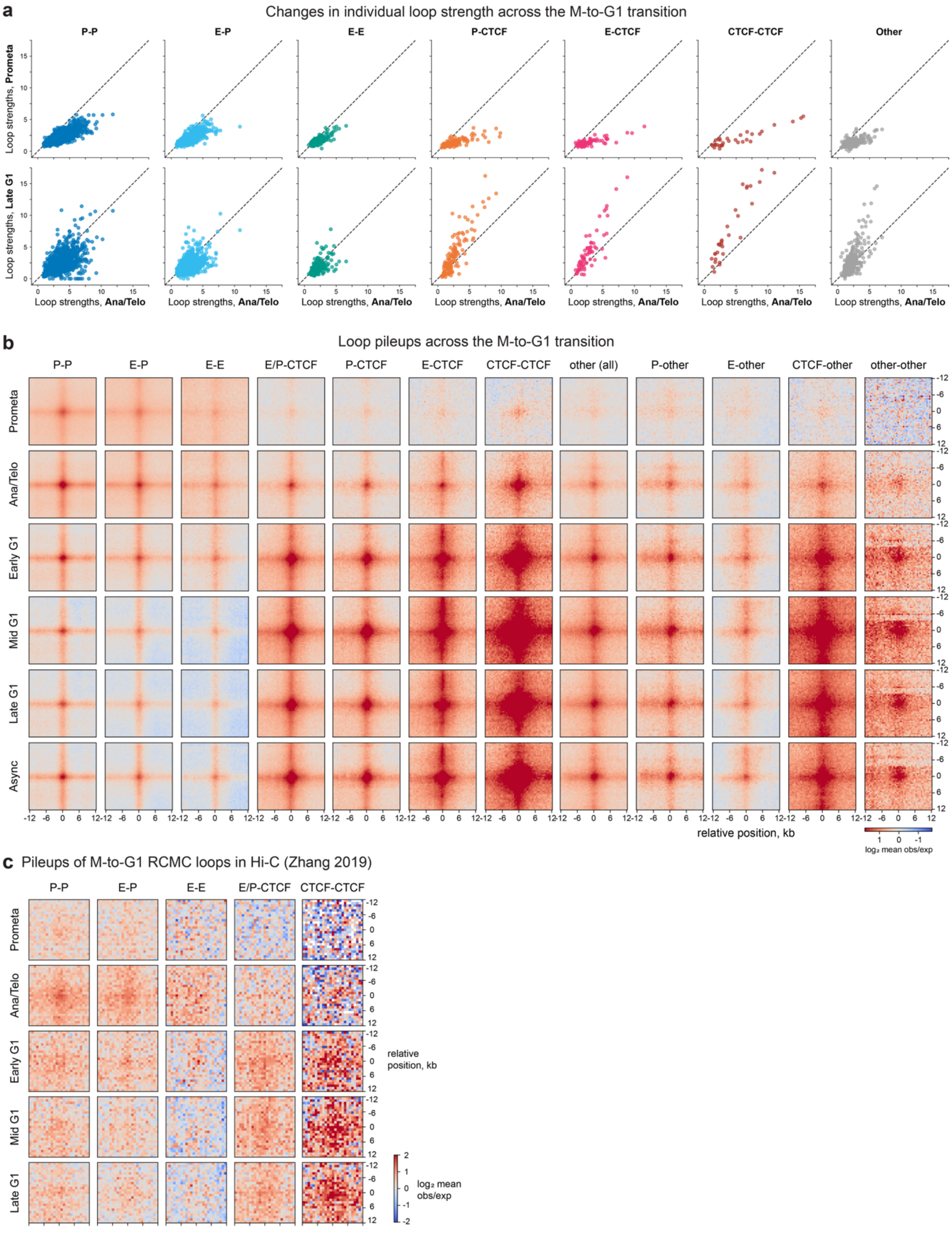

Supplementary Figure 3. Quantification of M-to-G1 RCMC experiments and loop strengths. (**a**) Plots of individual loop strengths of each loop category in Fig. 2e for the prometaphase (top) and late G1 (bottom) conditions, plotted against the strengths in the ana/telophase condition (x-axes). Strengths are calculated as the integrated observed loop signal divided by the expected background signal from local *P(s)* curves. The local *P(s)* curve used in this "observed over expected" strength calculation is determined by the loop distance and the dataset's interaction decay curve. These panels show "pure" loops using exclusive loop categorizations, wherein loops anchored by both CREs and CTCF/RAD21 at a single site have been removed. (**b**) Expanded array of the aggregate peak analysis (APA) plots shown in Fig. 3b, separated to show various loop classifications across the M-to-G1 transition and for the asynchronous condition. Plots show a 24 kb window centered on the loop at 500 bp resolution, and the loops plotted here and in all subsequent panels follow the "exclusive" definition of loop identity as in 2g (CRE sites do not overlap with CTCF). (**c**) APA plots in the Zhang *et al.* (2019) Hi-C data of the RCMC-identified loops shown in Fig. 3b. Plots show a 24 kb window centered on the loop at 1 kb resolution.

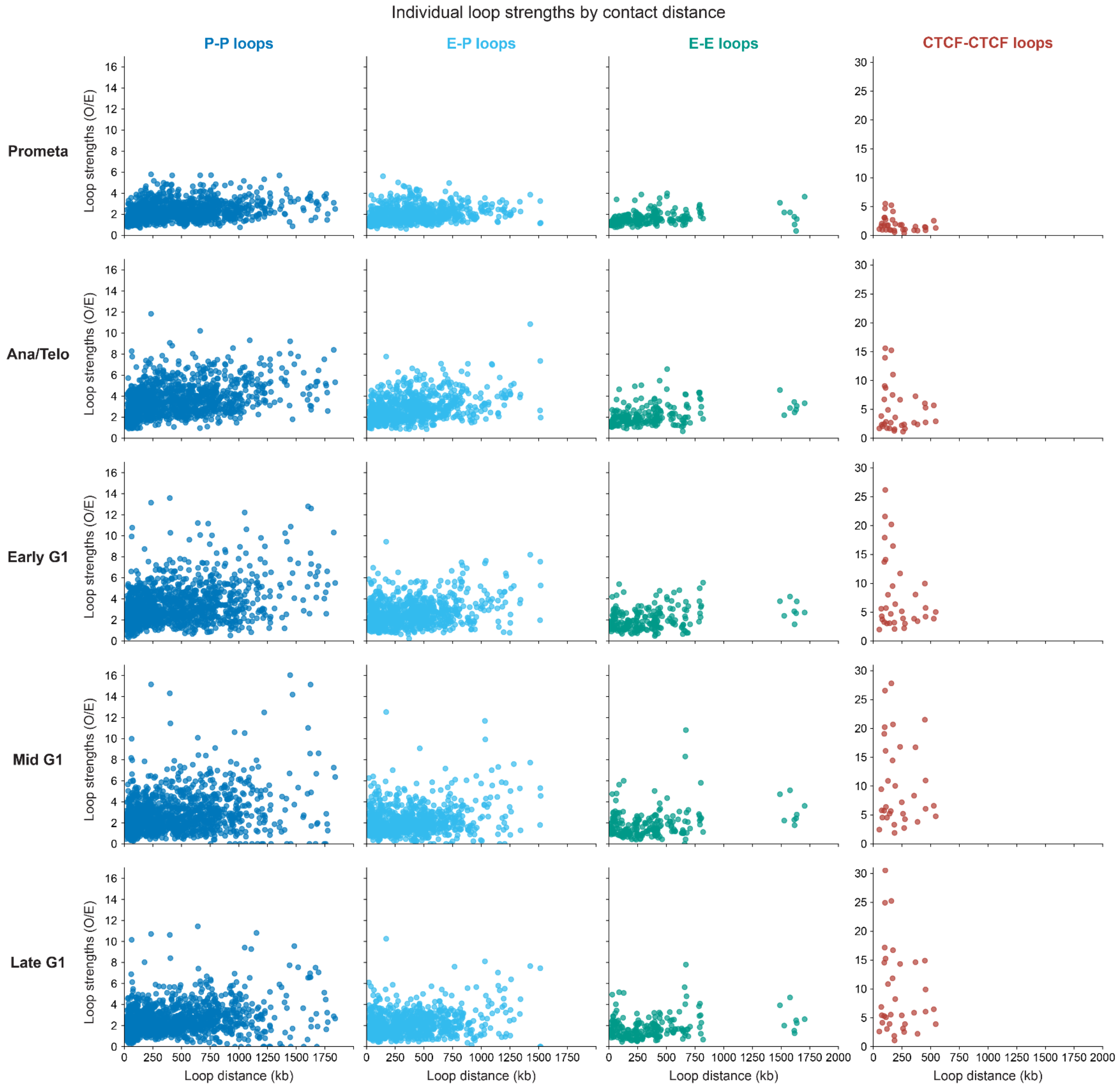

Supplementary Figure 4. Individual loop strengths by contact distance across the M-to-G1 transition. Plots of individual P-P (blue), E-P (light blue), E-E (green), and CTCF-CTCF (red) loop strengths (y-axes) plotted against loop size (x-axes) across the M-to-G1 transition. Strengths are calculated as the integrated observed loop signal divided by the expected background signal from local *P(s)* curves ("observed over expected"), and the "exclusive" loop categorization excludes loops that are both CREs and also CTCF/cohesin-bound.

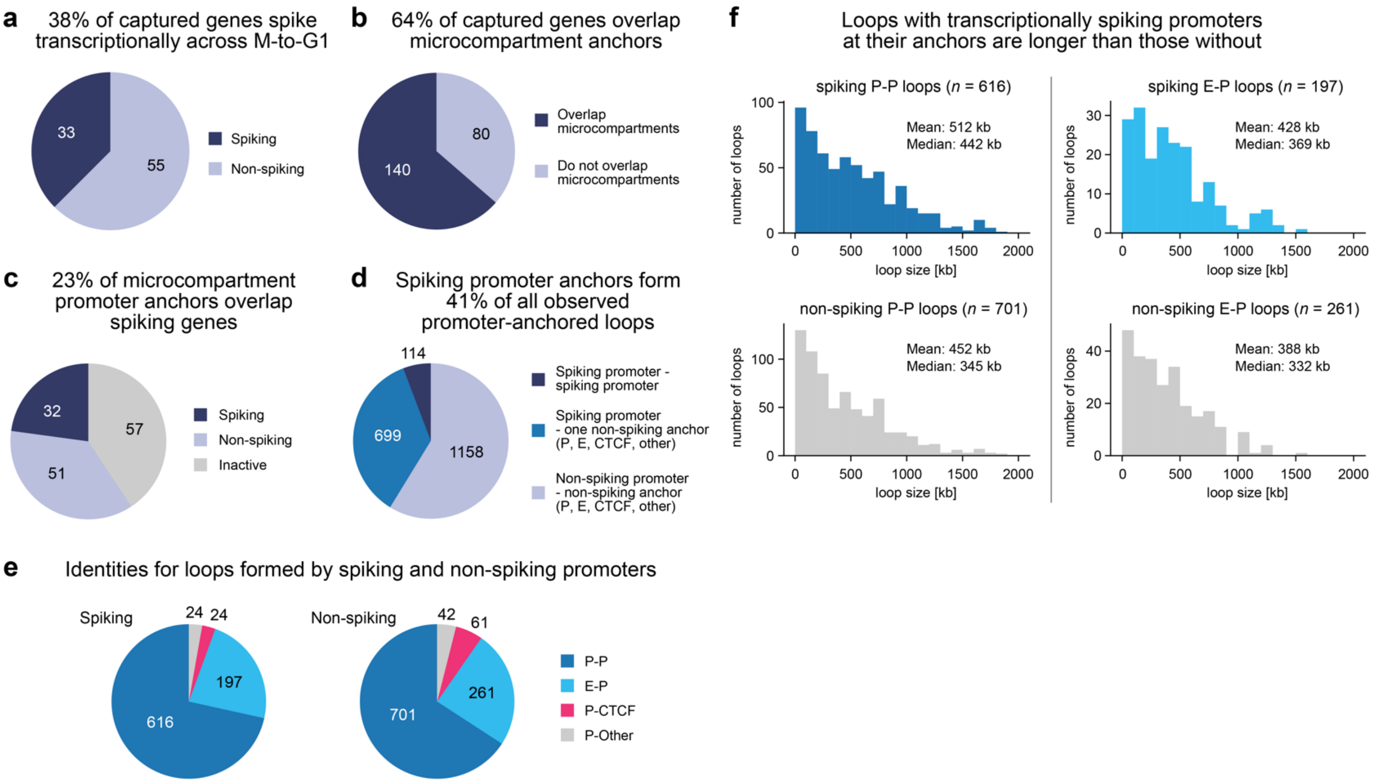

Supplementary Figure 5. Transcriptionally spiking genes form more loops and stronger loops. (a) The five regions captured using RCMC contain 88 unique genes identified to be active, 33 (38%) of which were identified to be transcriptionally spiking during the M-to-G1 transition. **(b)** The RCMC regions contain a total of 220 genes, 140 (64%) of which overlap microcompartment anchors. **(c)** Of the 140 microcompartment anchors identified as promoters across the RCMC regions, 32 (23%) of them overlap transcriptionally spiking genes. **(d)** The spiking promoter anchors identified in (c) form 114 loops with themselves and 699 loops with non-spiking anchors, while non-spiking promoter anchors form 1158 loops with other non-spiking anchors. The percentage of loops involving at least one spiking promoter is therefore 41%. **(e)** Pie chart of annotated loops by their functional identity, separated into loops containing a spiking promoter anchor (left) and those without a spiking promoter anchor (right). Promoters were identified as annotated transcription start sites ±2 kb, enhancers as non-promoter regions with overlapping H3K4me1 and H3K27ac ChIP-seq peaks, and CTCF/RAD21 as non-promoter and non-enhancer sites with overlapping CTCF and RAD21 ChIP-seq peaks. Anchors with multiple overlapping genomic features were hierarchically classified into a single classification, with promoters taking precedence, then enhancers, and finally CTCF/RAD21. Anchors designated as “other” do not overlap promoters, enhancers, nor CTCF/RAD21. P designates promoters, E designates enhancers, and CTCF designates CTCF/RAD21. **(f)** Histograms of loop interaction distances, separated by loop identity. P-P loops formed by one or two transcriptionally spiking promoters are in the top left, with P-P loops formed by two non-spiking promoters below. E-P loops formed by one spiking promoter and an enhancer are in the top right, with E-P loops formed by one non-spiking promoter and an enhancer below.

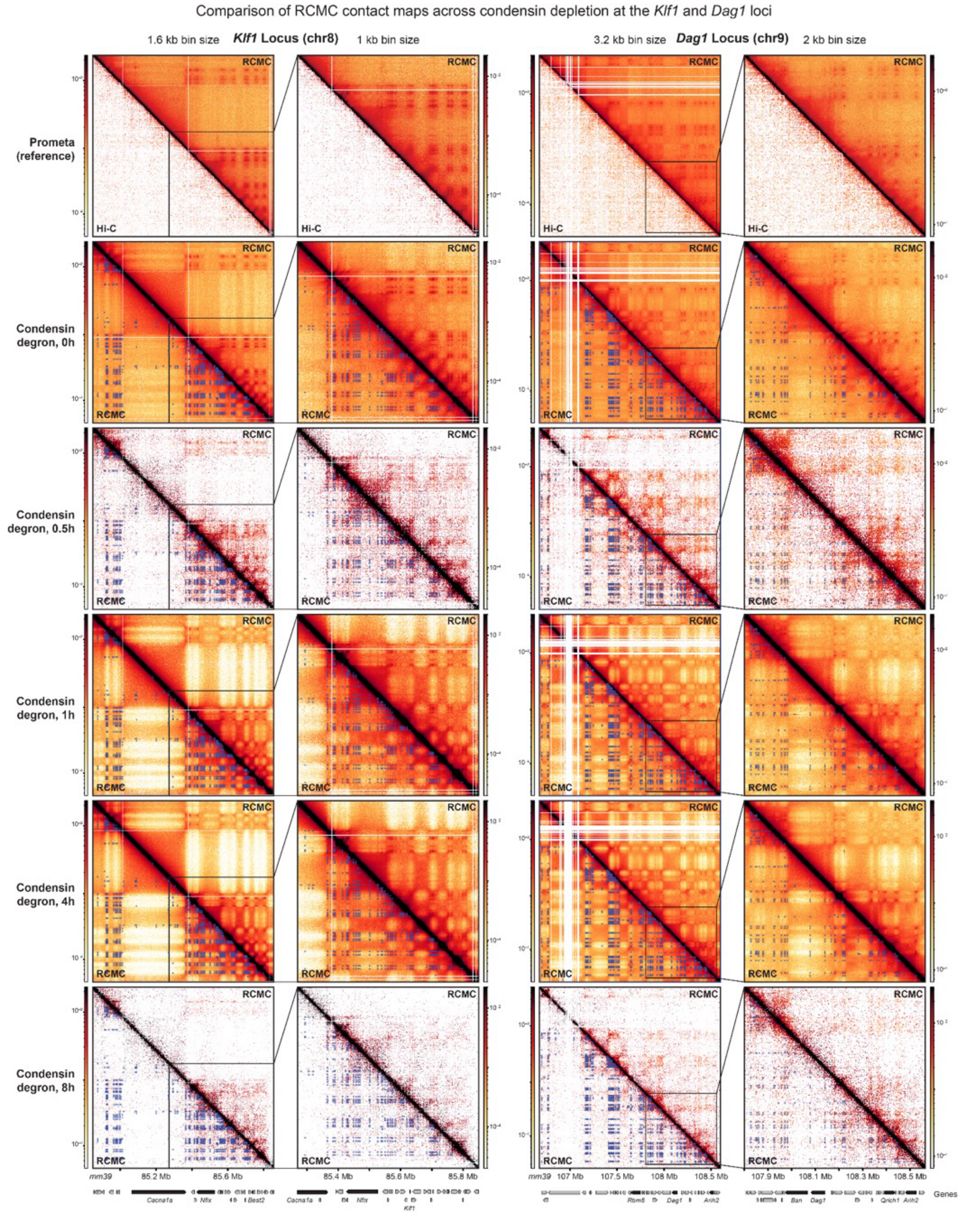

Supplementary Figure 6. RCMC maps condensin depletion at the *Klf1* and *Dag1* loci. RCMC contact map comparisons of increasing condensin degradation in prometaphase-arrested cells at the *Klf1* and *Dag1* loci. Condensin degradation of nocodazole-treated SMC2-mAID cells was induced using auxin treatments of 0.5h, 1h, 4h, and 8h, compared against a no treatment (0h) control. Input cell material for RCMC for the 0h, 1h, and 4h conditions was significantly greater than for the 0.5h and 8h conditions. Full capture regions are shown for both loci at 1.6 kb and 3.2 kb resolution, respectively, along with zoom-ins at 1 kb and 2 kb resolution, respectively. The prometaphase M-to-G1 RCMC dataset is shown at the top against previously generated Hi-C data[30] for reference. Gene annotations are shown below the contact maps and signal intensity scales are shown next to the maps.

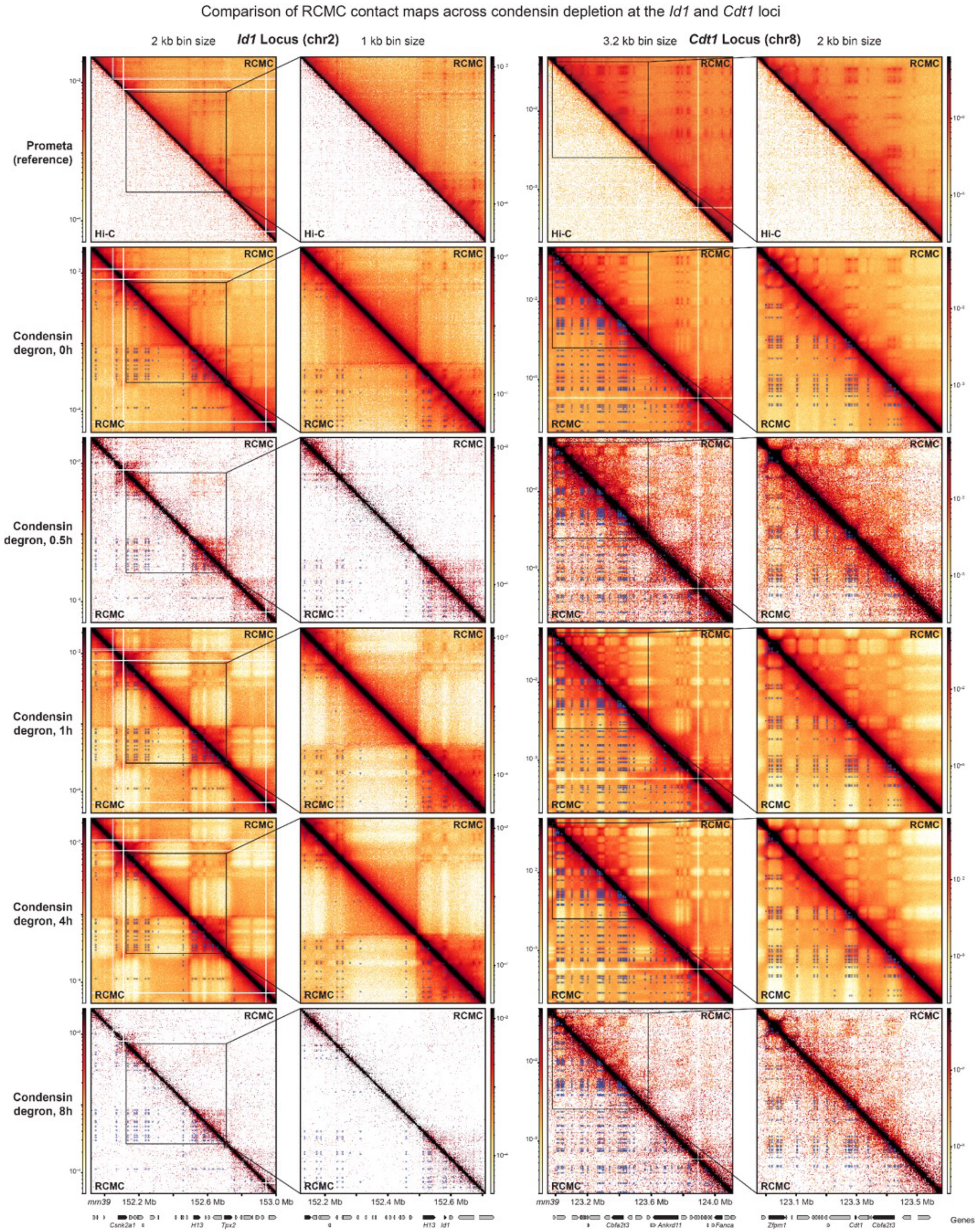

Supplementary Figure 7. RCMC maps condensin depletion at the *Id1* and *Cdt1* loci. RCMC contact map comparisons of increasing condensin degradation in prometaphase-arrested cells at the *Id1* and *Cdt1* loci. Condensin degradation of nocodazole-treated SMC2-mAID cells was induced using auxin treatments of 0.5h, 1h, 4h, and 8h, compared against a no treatment (0h) control. Input cell material for RCMC for the 0h, 1h, and 4h conditions was significantly greater than for the 0.5h and 8h conditions. Full capture regions are shown for both loci at 2 kb and 3.2 kb resolution, respectively, along with zoom-ins at 1 kb and 2 kb resolution, respectively. The prometaphase M-to-G1 RCMC dataset is shown at the top against previously generated Hi-C data[30] for reference. Gene annotations are shown below the contact maps and signal intensity scales are shown next to the maps.

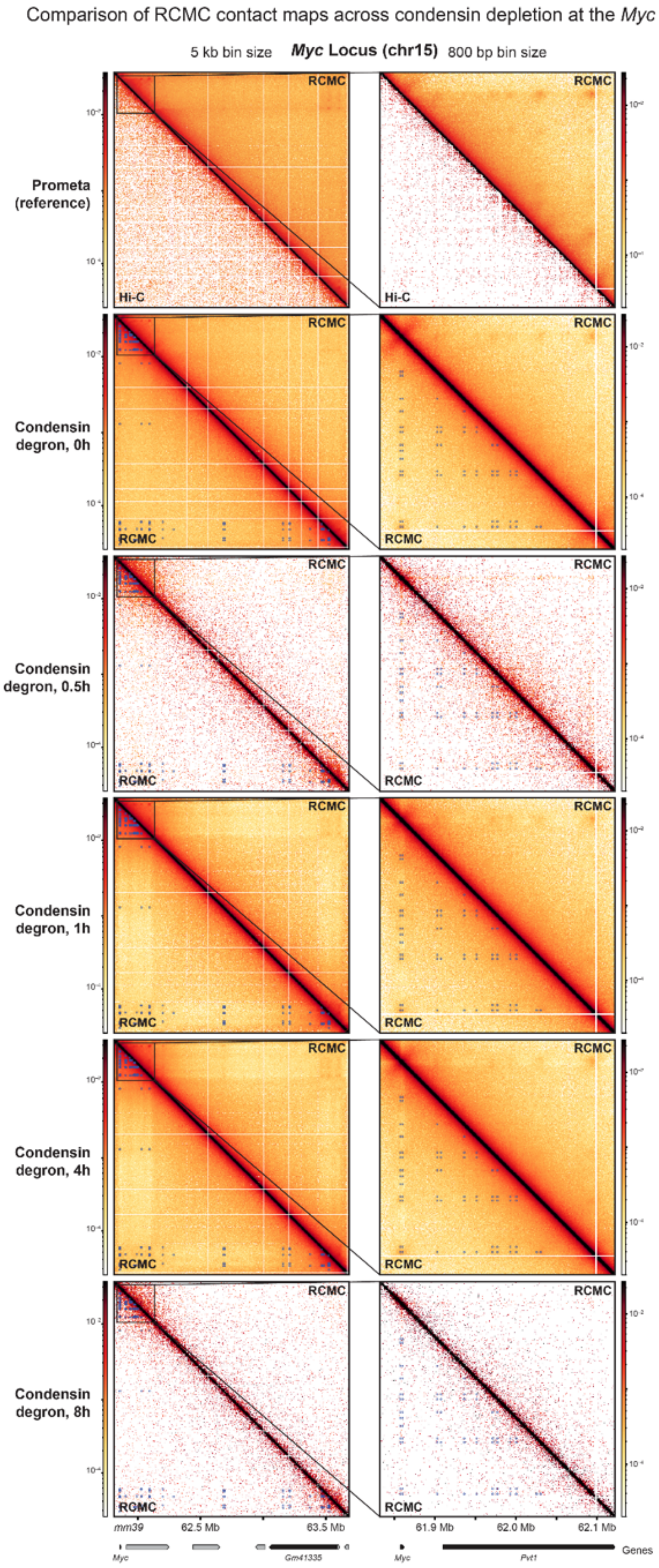

Supplementary Figure 8. RCMC maps condensin depletion at the *Myc* locus. RCMC contact map comparisons of increasing condensin degradation in prometaphase-arrested cells at the *Myc* locus. Condensin degradation of nocodazole-treated SMC2-mAID cells was induced using auxin treatments of 0.5h, 1h, 4h, and 8h, compared against a no treatment (0h) control. Input cell material for RCMC for the 0h, 1h, and 4h conditions was significantly greater than for the 0.5h and 8h conditions. The full capture region is shown at 5 kb resolution along with a zoom-in at 800 bp resolution. The prometaphase M-to-G1 RCMC dataset is shown at the top against previously generated Hi-C data[30] for reference. Gene annotations are shown below the contact maps and signal intensity scales are shown next to the maps.

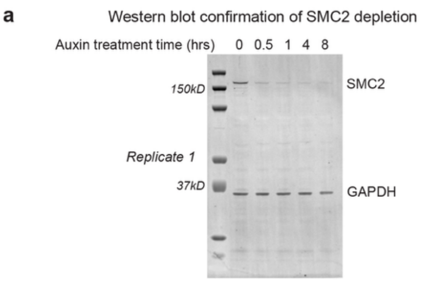

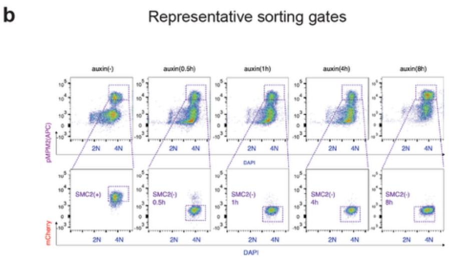

c Breakdown of SMC2 depletion sequencing depth

Read counts comparison	Unique sequencing reads genome-wide	Unique sequencing reads entirely in ROIs
RCMC, SMC2 depletion, **0h**	656,234,473	191,662,327
RCMC, SMC2 depletion, **0.5h***	67,631,171	5,609,551
RCMC, SMC2 depletion, **1h**	520,456,679	146,929,186
RCMC, SMC2 depletion, **4h**	598,908,999	199,379,034
RCMC, SMC2 depletion, **8h***	34,862,279	1,988,799

d SMC2 depletion RCMC cell inputs

Cell inputs by replicate	Rep1	Rep2	Rep3	Rep4	**Total**
RCMC, SMC2 depletion, **0h**	-	1.7M	6.9M	5.2M	13.8M
RCMC, SMC2 depletion, **0.5h**	1.7M	2.4M	-	-	4.1M
RCMC, SMC2 depletion, **1h**	1.2M	2.5M	5.0M	4.2M	12.9M
RCMC, SMC2 depletion, **4h**	0.2M	2.1M	2.1M	4.7M	9.1M
RCMC, SMC2 depletion, **8h**	0.6M	1.6M	-	-	2.2M

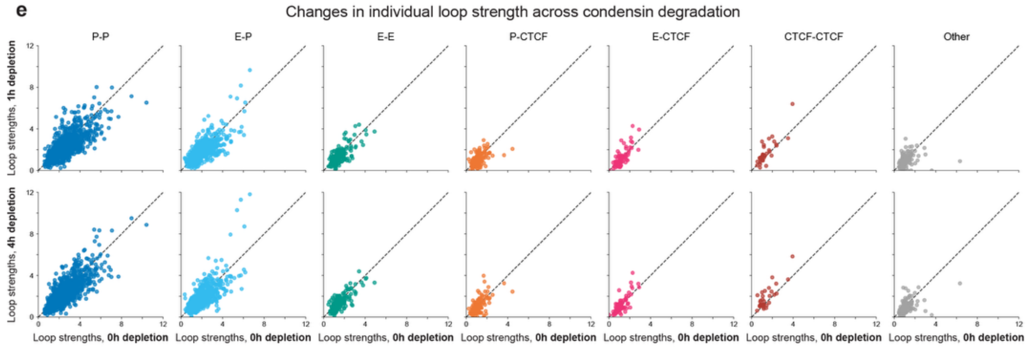

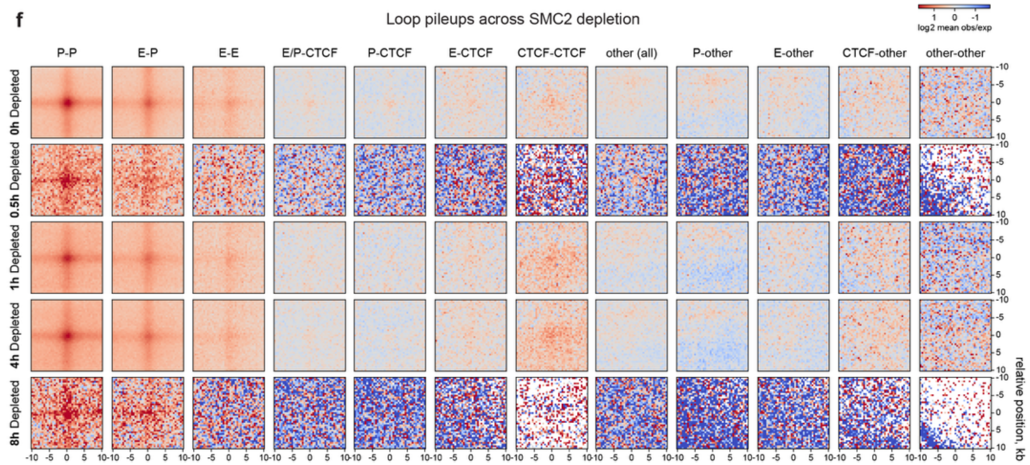

Supplementary Figure 9. Quantification of SMC2 depletion RCMC experiments and loop strengths. (**a**) Western blot confirmation of SMC2 depletion following treatment by auxin, with GAPDH serving as a loading control. Blots are shown for Replicate 1; Western blots were not run for Replicate 2. Quantification of depletion reveals 76% SMC2 depletion following 0.5h of treatment and 81-88% depletion following 1-8h of treatment. (**b**) Sorting gates used for selection of SMC2-depleted cells via flow cytometry following auxin treatment. Collecting the cell population low in mCherry ensures collection of cells with efficient degradation of SMC2 and exclusion of the small population of cells with inefficient degradation. (**c**) Table of uniquely mapped RCMC reads genome-wide and within captured ROIs for the five condensin depletion timepoints. Asterisks for the 0.5h and 8h conditions denote much shallower sequencing depths resulting from lower cell inputs. (**d**) Table of condensin-depleted cell sample inputs for RCMC, separated by condition and replicate. (**e**) Plots of individual loop strengths of each loop category in Fig. 2e for the 1h (top) and 4h (bottom) condensin depletion conditions, plotted against the strengths in the 0h condition (x-axes). Strengths are calculated as the integrated observed loop signal divided by the expected background signal from local *P(s)* curves. The local *P(s)* curves used in this “observed over expected” strength calculation are determined by the loop distance and the dataset’s interaction decay curve. These panels show “pure” loops using exclusive loop categorizations, wherein loops anchored by both CREs and CTCF/RAD21 at a single site have been removed. (**f**) Expanded array of the aggregate peak analysis (APA) plots shown in Fig. 5d, separated to show various loop classifications across condensin depletion. Plots show a 20 kb window centered on the loop at 500 bp resolution, and the loops plotted here and in all subsequent panels follow the “exclusive” definition of loop identity as in 2g (CRE sites do not overlap with CTCF).

a

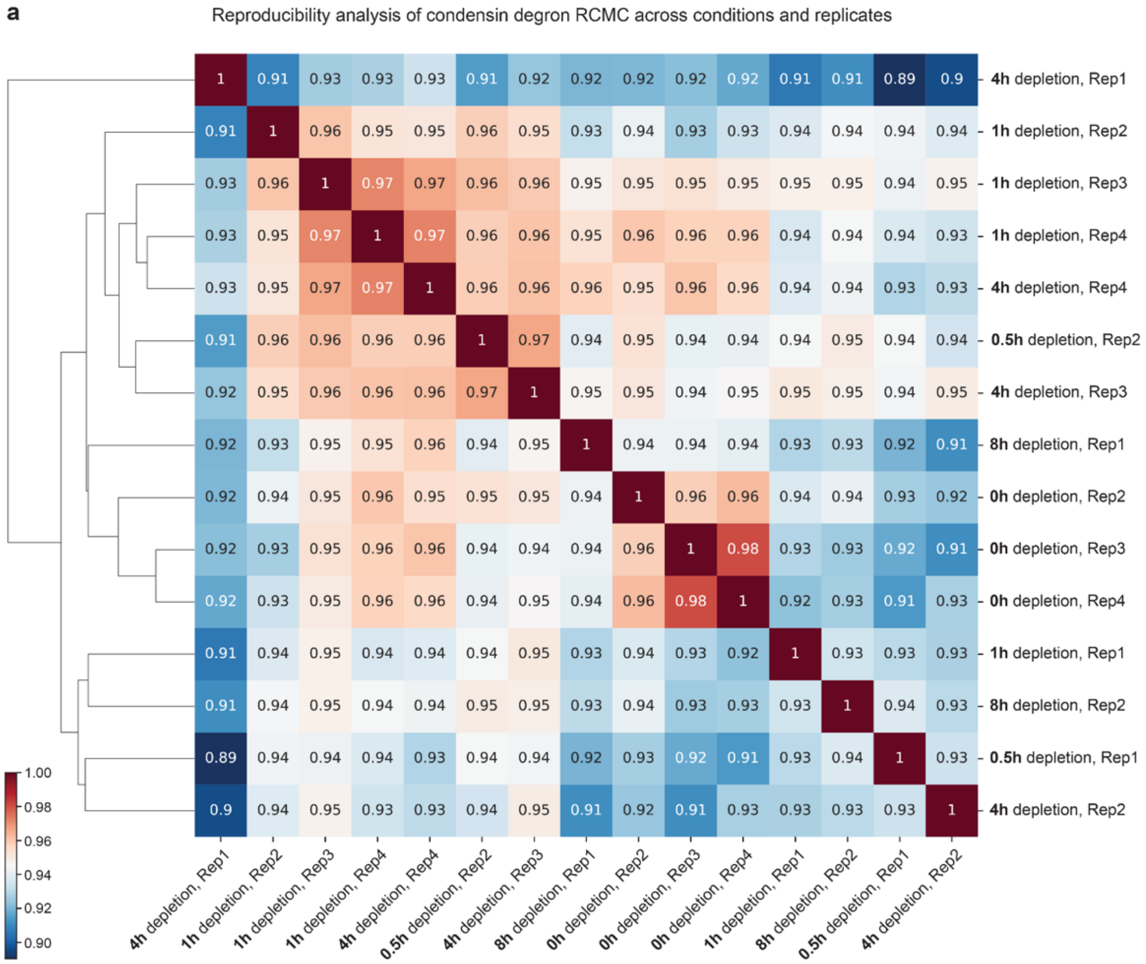

b Unique reads across SMC2 depletion RCMC samples by condition and replicate

	Rep1	Rep2	Rep3	Rep4	**Total**	
RCMC, SMC2 depletion, **0h**	-	1,063,941	113,798,631	76,799,755	191,662,327	>100M
RCMC, SMC2 depletion, **0.5h**	1,021,022	4,588,753	-	-	5,609,551	10-100M
RCMC, SMC2 depletion, **1h**	633,026	5,028,084	43,550,381	97,717,471	146,929,186	1-10M
RCMC, SMC2 depletion, **4h**	344,489	2,249,551	1,837,919	194,946,851	199,379,034	<1M
RCMC, SMC2 depletion, **8h**	482,762	1,506,261	-	-	1,988,799	

Supplementary Figure 10. Quantification of SMC2 degron RCMC experimental reproducibility and replicate depth. (**a**) Measurement of reproducibility between condensin depletion RCMC samples across conditions and replicates. Reproducibility scores are determined using HiCRep[31] at 5 kb resolution, averaged across all five captured loci, and clustered according to similarity. (**b**) Table of uniquely mapped RCMC reads across captured ROIs for the five condensin depletion timepoints, shown for each replicate. Values are color-coded to highlight magnitudes of difference in sequencing depth across samples, with a legend to the right of the table. Variability in library complexity and sequencing depth stems from the variability in cell inputs shown in Supplementary Figure 9d, with higher cellular inputs yielding more complex Micro-C libraries that became larger fractions of the captured RCMC libraries and were ultimately more deeply sequenced.

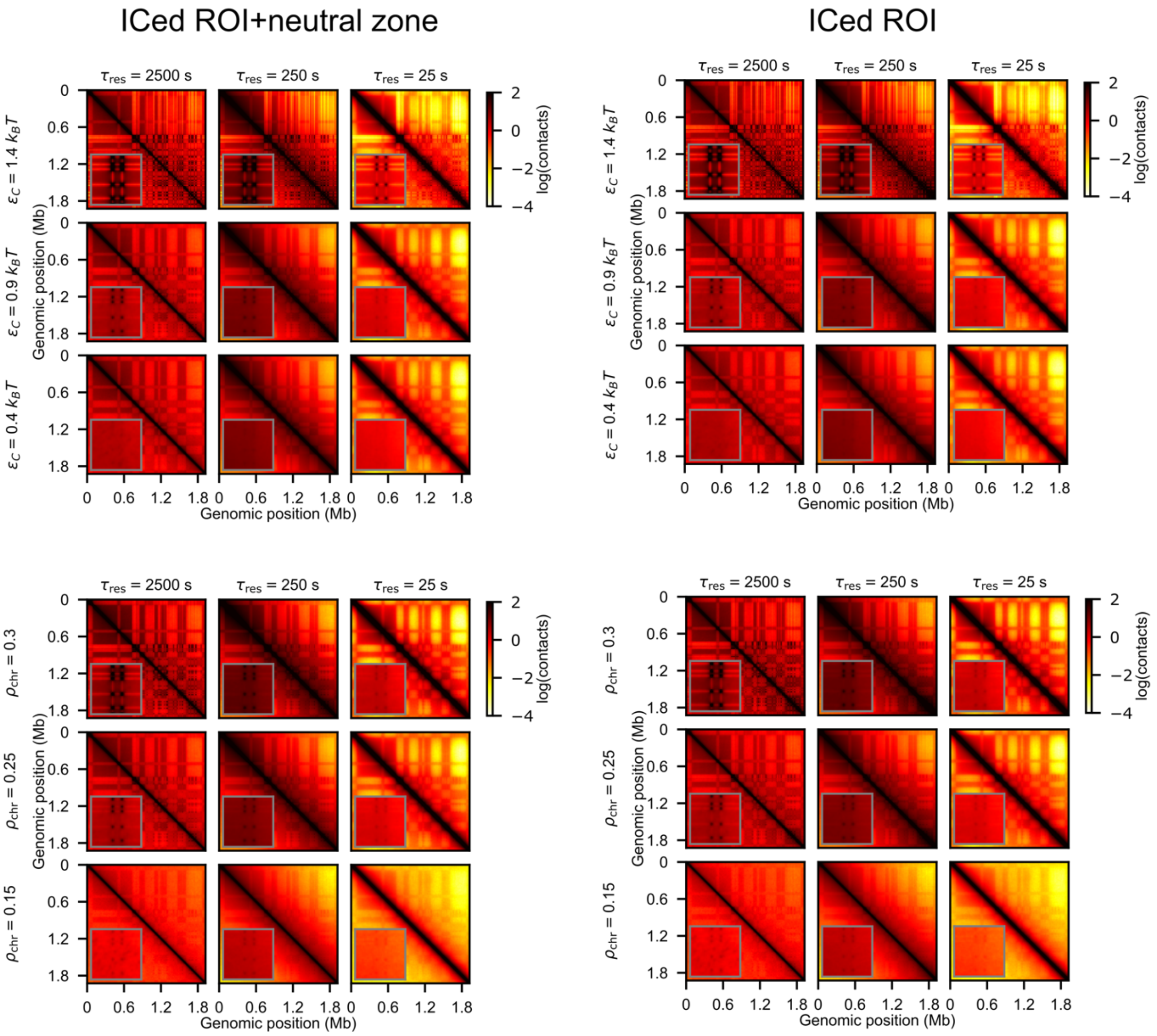

Supplementary Figure 11. Simulation parameter sweeps of different microcompartment affinities, extruder residence times, and chromatin densities with different contact map normalizations. Contact maps from steady-state simulations for the region of interest (ROI), *Dag1*, are displayed, with insets showing zoom-ins of microcompartments. *Left*: Contact maps of *Dag1*, with iterative correction (IC) applied to *Dag1* and the connected neutral polymer segment (not shown) together. *Right:* Contact maps of *Dag1*, with iterative correction (IC) applied to *Dag1* only. Top sets of maps show sweeps of microcompartment affinities, ϵ_C, and extruder residence times, τ_{res}. Bottom sets of maps show sweeps for chromatin densities, ρ_{chr}, and extruder residence times, τ_{res}.

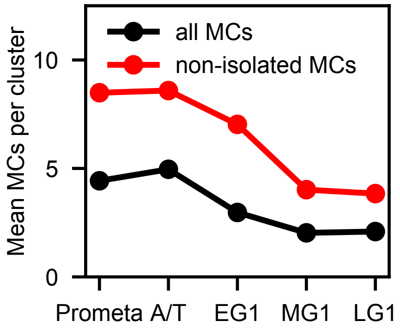

Supplementary Figure 12. Simulated microcompartments form multi-way contacts. Number of microcompartments from the local simulated *Dag1* repeat per microcompartment cluster throughout the M-to-G1 transition, averaged over single configurations from 10 different simulations. Data is plotted for all microcompartment clusters (black), which includes clusters with a single microcompartment anchor (*i.e.*, microcompartment anchors not contacting another microcompartment anchor), and non-isolated microcompartment clusters (red), which includes only those microcompartment clusters composed of more than one microcompartment anchor. Error bars (s.e.m.) are the size of the data points or smaller.

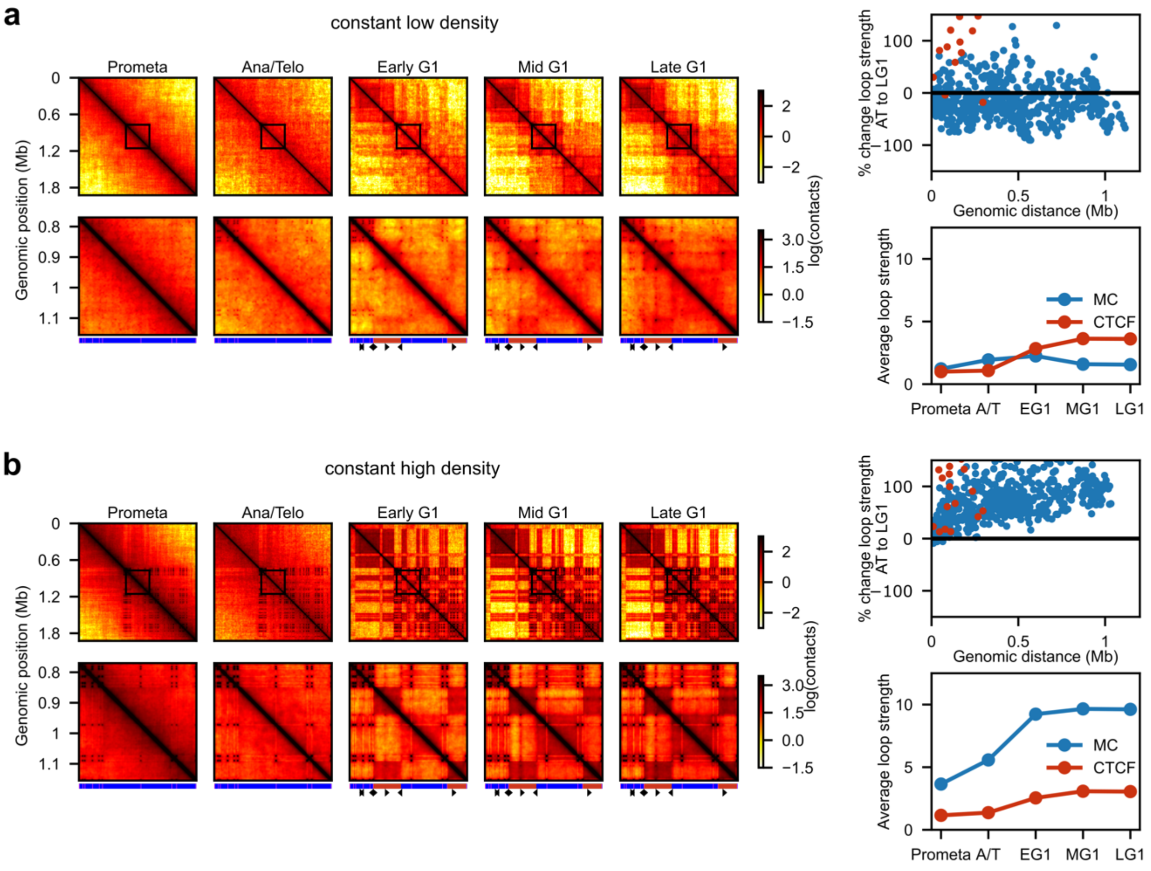

Supplementary Figure 13. Simulations of the mitosis-to-G1 transition with constant chromatin polymer density. Results from simulations of the mitosis-to-G1 with density held constant at either (**a**) ρ_{chr}=0.25 ("low") or (**b**) ρ_{chr}=0.65 ("high"). Left panels show contact maps from various times with the top row showing the full *Dag1* region and the bottom row showing a zoomed-in view of the region marked by the box in the top row. Compartment structure and CTCF sites are indicated below. Right panels show quantification of percent change in loop strength of simulated microcompartments from ana/telophase to late G1 as a function of loop size (top) and average microcompartment and CTCF loop strengths (bottom) throughout the mitosis-to-G1 transition.

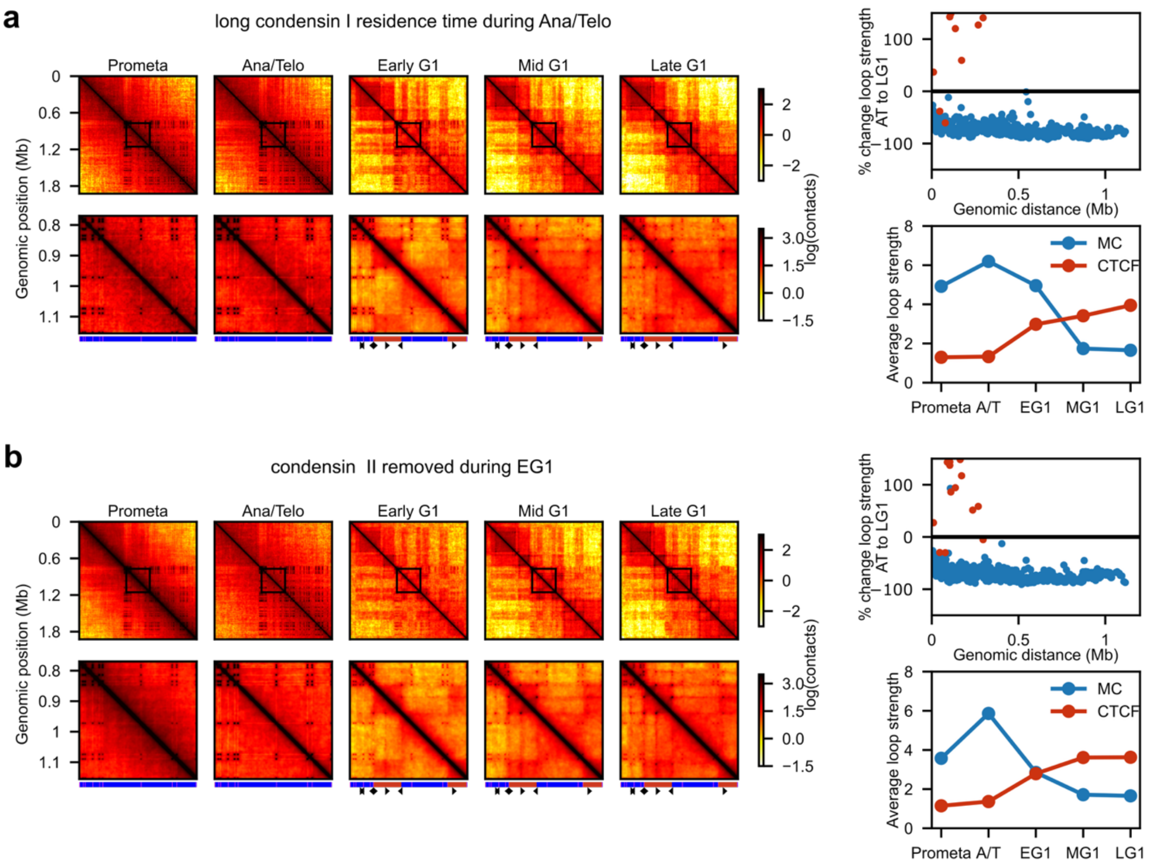

Supplementary Figure 14. Simulations of the mitosis-to-G1 transition with alterations to condensin I removal timing or ana/telophase turnover. Results from simulations of the mitosis-to-G1 with (**a**) condensin I removal during ana/telophase with residence time of condensin I, τ_{res}^{CI}, increased 20-fold (such that $\tau_{res}^{CI} = \tau_{res}^{CII}$) during ana/telophase and (**b**) condensin II occurring gradually during removal during the first 30 minutes of G1, rather than instantaneously at the onset of G1. Left panels show contact maps from various times with the top row showing the full *Dag1* region and the bottom row showing a zoomed-in view of the region marked by the box in the top row. Compartment structure and CTCF sites are indicated below. Right panels show quantification of percent change in loop strength of simulated microcompartments from ana/telophase to late G1 as a function of loop size (top) and average microcompartment and CTCF loop strengths (bottom) throughout the mitosis-to-G1 transition.

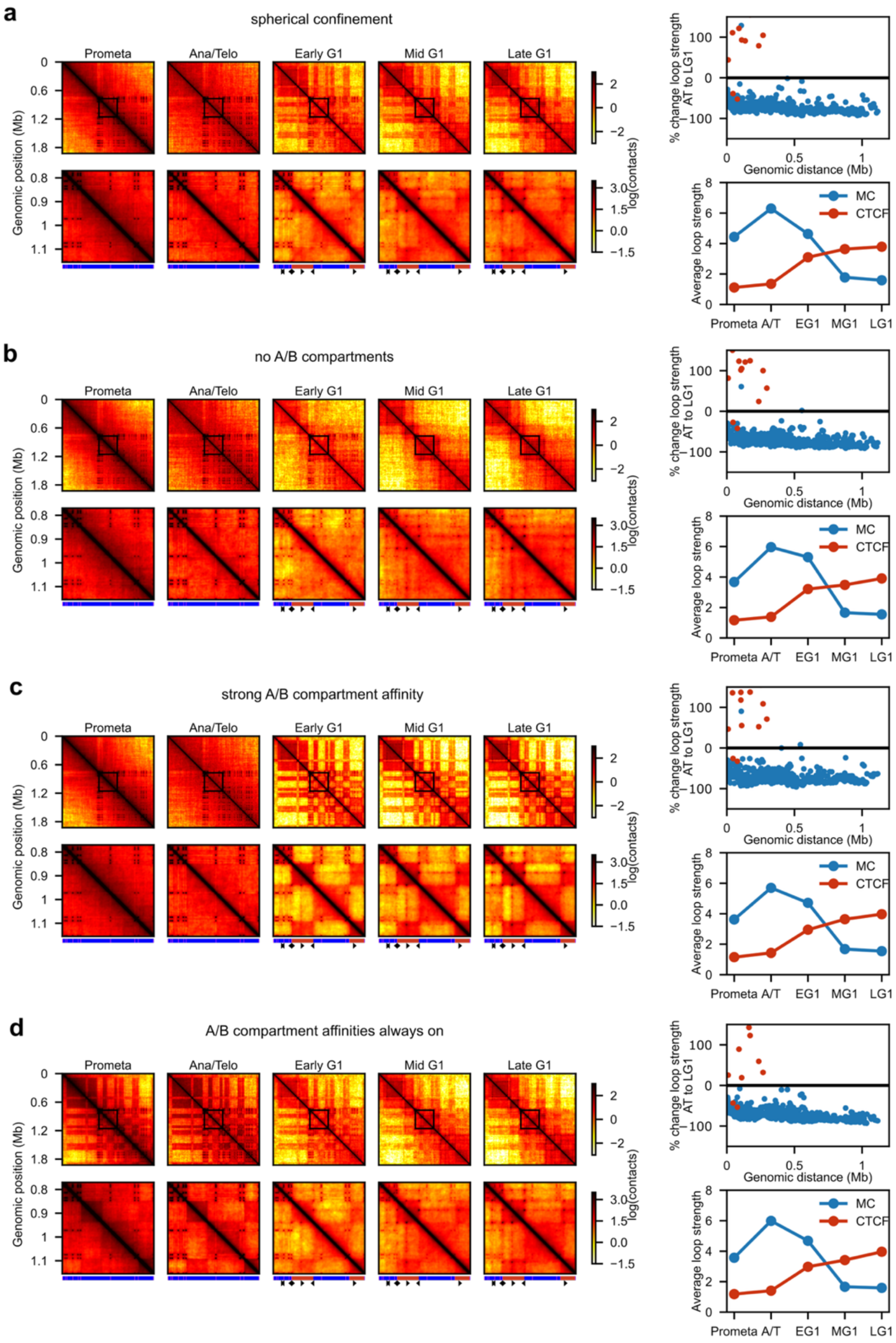

Supplementary Figure 15. Simulations of the mitosis-to-G1 transition with different boundary conditions or A/B compartment affinities. Results from simulations of the mitosis-to-G1 with (**a**) no cylindrical boundary condition during mitosis, i.e., only spherical confinement, such that while chromosome density changes, chromosome shape does not; (**b**) no A/B compartment affinity interactions; (**c**) A/B compartment affinity doubled to ϵ_B=0.1 k_BT; and (**d**) A/B compartment affinity always on at the typical value of ϵ_B=0.05 k_BT, including during prometaphase and ana/telophase. Left panels show contact maps from various times with the top row showing the full *Dag1* region and the bottom row showing a zoomed-in view of the region marked by the box in the top row. Compartment structure and CTCF sites are indicated below. Right panels show quantification of percent change in loop strength of simulated microcompartments from ana/telophase to late G1 as a function of loop size (top) and average microcompartment and CTCF loop strengths (bottom) throughout the mitosis-to-G1 transition.

Supplementary Information references

1. Goel, V. Y., Huseyin, M. K. & Hansen, A. S. Region Capture Micro-C reveals coalescence of enhancers and promoters into nested microcompartments. *Nat Genet* **55**, 1048–1056 (2023).
2. Gabriele, M. *et al.* Dynamics of CTCF- and cohesin-mediated chromatin looping revealed by live-cell imaging. *Science* **376**, 496–501 (2022).
3. Gerlich, D., Koch, B., Dupeux, F., Peters, J.-M. & Ellenberg, J. Live-Cell Imaging Reveals a Stable Cohesin-Chromatin Interaction after but Not before DNA Replication. *Current Biology* **16**, 1571–1578 (2006).
4. Gerlich, D., Hirota, T., Koch, B., Peters, J.-M. & Ellenberg, J. Condensin I stabilizes chromosomes mechanically through a dynamic interaction in live cells. *Curr Biol* **16**, 333–344 (2006).
5. Walther, N. *et al.* A quantitative map of human Condensins provides new insights into mitotic chromosome architecture. *Journal of Cell Biology* **217**, 2309–2328 (2018).
6. Brunner, A. *et al.* Quantitative imaging of loop extruders rebuilding interphase genome architecture after mitosis. 2024.05.29.596439 Preprint at https://doi.org/10.1101/2024.05.29.596439 (2024).
7. Kueng, S. *et al.* Wapl Controls the Dynamic Association of Cohesin with Chromatin. *Cell* **127**, 955–967 (2006).
8. Tedeschi, A. *et al.* Wapl is an essential regulator of chromatin structure and chromosome segregation. *Nature* **501**, 564–568 (2013).
9. Wutz, G. *et al.* ESCO1 and CTCF enable formation of long chromatin loops by protecting cohesinSTAG1 from WAPL. *eLife* **9**, e52091 (2020).
10. Hansen, A. S., Pustova, I., Cattoglio, C., Tjian, R. & Darzacq, X. CTCF and cohesin regulate chromatin loop stability with distinct dynamics. *eLife* **6**, e25776 (2017).
11. Cattoglio, C. *et al.* Determining cellular CTCF and cohesin abundances to constrain 3D genome models. *eLife* **8**, e40164 (2019).
12. Holzmann, J. *et al.* Absolute quantification of cohesin, CTCF and their regulators in human cells. *eLife* **8**, e46269 (2019).
13. Fudenberg, G. *et al.* Formation of Chromosomal Domains by Loop Extrusion. *Cell Reports* (2016) doi:10.1016/j.celrep.2016.04.085.
14. Ganji, M. *et al.* Real-time imaging of DNA loop extrusion by condensin. *Science* **360**, 102 LP – 105 (2018).
15. Davidson, I. F. *et al.* DNA loop extrusion by human cohesin. *Science* **366**, 1338 LP – 1345 (2019).
16. Kim, Y., Shi, Z., Zhang, H., Finkelstein, I. J. & Yu, H. Human cohesin compacts DNA by loop extrusion. *Science* **366**, 1345 LP – 1349 (2019).
17. Golfier, S., Quail, T., Kimura, H. & Brugués, J. Cohesin and condensin extrude DNA loops in a cell cycle-dependent manner. *eLife* **9**, e53885 (2020).
18. Kong, M. *et al.* Human Condensin I and II Drive Extensive ATP-Dependent Compaction of Nucleosome-Bound DNA. *Mol Cell* **79**, 99-114.e9 (2020).
19. Falk, M. *et al.* Heterochromatin drives compartmentalization of inverted and conventional nuclei. *Nature* **570**, 395–399 (2019).
20. Attar, A. G., Paturej, J., Banigan, E. J. & Erbaş, A. Chromatin phase separation and nuclear shape fluctuations are correlated in a polymer model of the nucleus. *Nucleus* **15**, 2351957 (2024).
21. Ou, H. D. *et al.* ChromEMT: Visualizing 3D chromatin structure and compaction in interphase and mitotic cells. *Science* **357**, eaag0025 (2017).
22. Gibcus, J. H. *et al.* A pathway for mitotic chromosome formation. *Science* **359**, eaao6135 (2018).
23. Takemoto, A., Kimura, K., Yokoyama, S. & Hanaoka, F. Cell cycle-dependent phosphorylation, nuclear localization, and activation of human condensin. *J Biol Chem* **279**, 4551–4559 (2004).
24. Fukui, K. & Uchiyama, S. Chromosome protein framework from proteome analysis of isolated human metaphase chromosomes. *Chem Rec* **7**, 230–237 (2007).
25. Shintomi, K. & Hirano, T. The relative ratio of condensin I to II determines chromosome shapes. *Genes Dev* **25**, 1464–1469 (2011).
26. Ono, T. *et al.* Differential contributions of condensin I and condensin II to mitotic chromosome architecture in vertebrate cells. *Cell* **115**, 109–121 (2003).
27. Cisneros-Soberanis, F. *et al.* Near millimolar concentration of nucleosomes in mitotic chromosomes from late prometaphase into anaphase. *Journal of Cell Biology* **223**, e202403165 (2024).
28. Mora-Bermúdez, F., Gerlich, D. & Ellenberg, J. Maximal chromosome compaction occurs by axial shortening in anaphase and depends on Aurora kinase. *Nat Cell Biol* **9**, 822–831 (2007).
29. Hildebrand, E. M. *et al.* Mitotic chromosomes are self-entangled and disentangle through a topoisomerase-II-dependent two-stage exit from mitosis. *Mol Cell* **84**, 1422-1441.e14 (2024).
30. Zhang, H. *et al.* Chromatin structure dynamics during the mitosis-to-G1 phase transition. *Nature* **576**, 158–162 (2019).
31. Zhao, H. *et al.* Genome folding principles uncovered in condensin-depleted mitotic chromosomes. *Nat Genet* **56**, 1213–1224 (2024).
