# Supplementary material for: Dynamics of microcompartment formation at the mitosis-to-G1 transition
Source: Nat Struct Mol Biol. 2025 Oct 17;32(12):2614–27. doi: 10.1038/s41594-025-01687-2 (PMC12700819; doi:10.1038/s41594-025-01687-2)
Supplement: Supplementary file 2 — Reporting Summary [file 41594_2025_1687_MOESM2_ESM.pdf]

nature portfolio

Corresponding author(s): Edward J. Banigan, Anders S. Hansen

Last updated by author(s): May 22, 2025

Reporting Summary

Nature Portfolio wishes to improve the reproducibility of the work that we publish. This form provides structure for consistency and transparency in reporting. For further information on Nature Portfolio policies, see our Editorial Policies and the Editorial Policy Checklist.

Statistics

For all statistical analyses, confirm that the following items are present in the figure legend, table legend, main text, or Methods section.

n/a	Confirmed	
☐	☒	The exact sample size (*n*) for each experimental group/condition, given as a discrete number and unit of measurement
☐	☒	A statement on whether measurements were taken from distinct samples or whether the same sample was measured repeatedly
☒	☐	The statistical test(s) used AND whether they are one- or two-sided<br>*Only common tests should be described solely by name; describe more complex techniques in the Methods section.*
☒	☐	A description of all covariates tested
☒	☐	A description of any assumptions or corrections, such as tests of normality and adjustment for multiple comparisons
☐	☒	A full description of the statistical parameters including central tendency (e.g. means) or other basic estimates (e.g. regression coefficient) AND variation (e.g. standard deviation) or associated estimates of uncertainty (e.g. confidence intervals)
☒	☐	For null hypothesis testing, the test statistic (e.g. *F*, *t*, *r*) with confidence intervals, effect sizes, degrees of freedom and *P* value noted<br>*Give P values as exact values whenever suitable.*
☒	☐	For Bayesian analysis, information on the choice of priors and Markov chain Monte Carlo settings
☒	☐	For hierarchical and complex designs, identification of the appropriate level for tests and full reporting of outcomes
☒	☐	Estimates of effect sizes (e.g. Cohen's *d*, Pearson's *r*), indicating how they were calculated

Our web collection on statistics for biologists contains articles on many of the points above.

Software and code

Policy information about availability of computer code

Data collection	No software was used for data collection. RCMC analysis code is available on GitHub at https://github.com/ahansenlab/RCMC_mitosis_analysis_code and polymer simulation code is also available on GitHub at https://github.com/mirnylab/microcompartments
Data analysis	bcl2fastq v2.20.0.422, fastqc v0.11.9, bwa-mem2 v2.2.1, pairtools v0.3.0, pairix v0.3.7, cooler v0.8.11, higlass v0.8.0, cooltools v0.5.0, coolbox v0.3.3, crossmap v0.6.1, IGV v2.10.3, hicrep v1.12.2, mustache v1.2.4, bigWigToBedGraph v377, MACS2 v2.2.7.1, FIMO v5.4.1, bedtools v2.30.0, R v4.1.2, deeptools v3.5.1, Python 3.7.12, Python 3.11.5 (simulations), polychrom v0.1.0, OpenMM v8.1.0<br>RCMC analysis code is available on GitHub at https://github.com/ahansenlab/RCMC_mitosis_analysis_code and polymer simulation code is also available on GitHub at https://github.com/mirnylab/microcompartments

For manuscripts utilizing custom algorithms or software that are central to the research but not yet described in published literature, software must be made available to editors and reviewers. We strongly encourage code deposition in a community repository (e.g. GitHub). See the Nature Portfolio guidelines for submitting code & software for further information.

Data

Policy information about availability of data

All manuscripts must include a data availability statement. This statement should provide the following information, where applicable:

- Accession codes, unique identifiers, or web links for publicly available datasets
- A description of any restrictions on data availability
- For clinical datasets or third party data, please ensure that the statement adheres to our policy

Sequencing data is available at NCBI Gene Expression Omnibus under accession number GSE276657 at https://www.ncbi.nlm.nih.gov/geo/query/acc.cgi?acc=GSE276657

Research involving human participants, their data, or biological material

Policy information about studies with human participants or human data. See also policy information about sex, gender (identity/presentation), and sexual orientation and race, ethnicity and racism.

Reporting on sex and gender	Not applicable.
Reporting on race, ethnicity, or other socially relevant groupings	Not applicable.
Population characteristics	Not applicable.
Recruitment	Not applicable.
Ethics oversight	Not applicable.

Note that full information on the approval of the study protocol must also be provided in the manuscript.

Field-specific reporting

Please select the one below that is the best fit for your research. If you are not sure, read the appropriate sections before making your selection.

☒ Life sciences ☐ Behavioural & social sciences ☐ Ecological, evolutionary & environmental sciences

For a reference copy of the document with all sections, see nature.com/documents/nr-reporting-summary-flat.pdf

Life sciences study design

All studies must disclose on these points even when the disclosure is negative.

Sample size	Sample sizes were determined based on the sample sizes chosen for similar experiments and analyses using other chromosome conformation capture techniques. Specifically, prior Micro-C (e.g., Hsieh et al., (2020)) and Capture (e.g., Goel et al., (2023)) studies reflected the cellular inputs required to achieve sufficient library complexity for fine-scale genome architecture mapping as well as the sequencing depth necessary to resolve structures.
Data exclusions	No data were excluded.
Replication	All RCMC maps in this study were highly reproducible. Specifically, reproducibility was determined quantitatively using HiCRep as shown in the manuscript.
Randomization	Not applicable.
Blinding	Not applicable.

Reporting for specific materials, systems and methods

We require information from authors about some types of materials, experimental systems and methods used in many studies. Here, indicate whether each material, system or method listed is relevant to your study. If you are not sure if a list item applies to your research, read the appropriate section before selecting a response.

Materials & experimental systems

n/a	Involved in the study
☒	☐ Antibodies
☐	☒ Eukaryotic cell lines
☒	☐ Palaeontology and archaeology
☒	☐ Animals and other organisms
☒	☐ Clinical data
☒	☐ Dual use research of concern
☒	☐ Plants

Methods

n/a	Involved in the study
☒	☐ ChIP-seq
☐	☒ Flow cytometry
☒	☐ MRI-based neuroimaging

Eukaryotic cell lines

Policy information about cell lines and Sex and Gender in Research

Cell line source(s)

The G1E-ER4 cell line was a gift from the Mitchell J. Weiss laboratory at St. Jude Children's Hospital. The SMC2-AID G1E-ER4 subline was previously generated and reported (Zhao et al., (2024)), and was provided by coauthor Haoyue Zhang.

Authentication

We regularly confirm that these cells can be induced to undergo terminal erythroid differentiation. All cell lines utilized in this study have previously been validated by PCR, sequencing, and western blotting, and by fluorescence microscopy where appropriate.

Mycoplasma contamination

G1E-ER4 cells have been tested to be negative for mycoplasma.

Commonly misidentified lines
(See ICLAC register)

No commonly misidentified cell lines were used.

Plants

Seed stocks

Not applicable.

Novel plant genotypes

Not applicable.

Authentication

Not applicable.

Flow Cytometry

Plots

Confirm that:

☒ The axis labels state the marker and fluorochrome used (e.g. CD4-FITC).

☒ The axis scales are clearly visible. Include numbers along axes only for bottom left plot of group (a 'group' is an analysis of identical markers).

☒ All plots are contour plots with outliers or pseudocolor plots.

☒ A numerical value for number of cells or percentage (with statistics) is provided.

Methodology

Sample preparation

Please see the Methods section.

Instrument

Beckman Coulter MoFlo Astrios EQ sorter.

Software

FlowJo v.10.8.1.

Cell population abundance

We obtained generally obtain >95% viable cells based on FSC-A and SSC-A.

Gating strategy

To isolate pure cell populations in PM, AT, early G1, mid G1 and late G1, the following markers were used to isolate specific cell populations: prometaphase – high mCherry-MD, positive pMPM2, 4N DAPI, ana/telophase- low mCherry-MD, 4N DAPI, G1 populations- negative mCherry-MD, 2N DAPI. Sorted cells were aliquoted and flash frozen in liquid nitrogen.

To isolate pure cell populations before and after SMC2 degradation, cells were subjected to flow cytometry to enrich for prometaphase-arrested samples. All samples were sorted for pMPM2+ cells; auxin-treated cells were sorted based on low mCherry signal (indicative of SMC2 degradation). Please see methods section for the antibody dilutions.

☒ Tick this box to confirm that a figure exemplifying the gating strategy is provided in the Supplementary Information.
